# Anemia of inflammation in patients with colorectal cancer: Correlation with interleukin-1, interleukin-33 and galectin-1

**DOI:** 10.5937/jomb0-30135

**Published:** 2022-02-02

**Authors:** Miodrag Jocić, Nebojša Arsenijević, Nevena Gajović, Milena Jurišević, Ivan Jovanović, Milan Jovanović, Nataša Zdravković, Veljko Marić, Marina Jovanović

**Affiliations:** 1 Military Medical Academy, Institute for Transfusiology and Haemobiology; 2 University of Kragujevac, Faculty of Medical Sciences, Center for Molecular Medicine and Stem Cell Research; 3 University of Kragujevac, Faculty of Medical Sciences, Department of Clinical Pharmacy; 4 Military Medical Academy, Department of Abdominal Surgery; 5 University of Kragujevac, Faculty of Medical Sciences, Department of Internal Medicine; 6 University of East Sarajevo, Faculty of Medicine Foca, Department of Surgery, Bosnia and Herzegovina

**Keywords:** Anemia, Colorectal carcinoma, Gal-1, IL-1, IL-33, anemija, koloreklatni karcinom, GAL-1, IL-1, IL-33

## Abstract

**Background:**

Patients with colorectal cancer (CRC) have anemia often present as a consequence of chronic bleeding from tumor. The exact role of lL-33, Galectin-l and IL-l in the pathological genesis of anemia in colorectal cancer patients has not been elucidated yet. The main goal of this research was to analyze Gal-l, IL-l and lL-33 systemic values in anemic and non-anemic CRC patients.

**Methods:**

Concentrations of IL-33, Galectin-1 and IL-1 have been studied in blood samples of 55 CRC patients (27 without anemia and 28 with anemia).

**Results:**

CRC patients with anemia had more severe and local advanced disease compared to CRC non-anemic patients. Anemia positively correlated with higher nuclear grade, lymph and blood vessel invasion, as well as with higher TNM stage, detectable metastatic lesions in lung and liver and peritoneal carcinomatosis. Significantly higher IL-33, Gal-1 and IL-1 concentration have been found in sera of patients with CRC and detected anemia. CRC patients mostly had microcytic anemia, while ferritin values were in normal range. Analysis revealed positive mutual correlation between serum values of galectin-1, IL-1 and IL-33 in CRC patients. Level of hemoglobin negatively correlated with serum IL-33, Gal-1 and IL-1. We have analyzed the Receiver Operating Characteristic (ROC) curves of serum IL-33, Gal-1 and IL-1 showed that these cytokines can be treated as additional markers for anemia of inflammation in CRC patients.

**Conclusions:**

Predomination of Galectin-1, IL-1 and IL-33 in anemic CRC patients implicates on their potential role in anemia genesis and further development.

## Abstract (sr)

**Uvod:**

Pacijenti sa kolorektalnim karcinomom (CRC) imaju anemiju koja je često prisutna kao posledica hroničnog krvarenja iz tumora. Tačna uloga IL-33, galektin-1 i IL-1 u patološkoj genezi anemije kod pacijenata sa kolorektalnim karcinomom još nije razjašnjena. Glavni cilj ovog istraživanja bio je analiza sistemskih vrednosti Gal-1, IL-1 i lL-33 kod anemičnih i neanemičnih CRC pacijenata.

**Metode:**

Koncentracije IL-33, galektin-1 i IL-1 ispitivane su u uzorcima krvi 55 pacijenata sa CRC (27 bez anemije i 28 sa anemijom).

**Rezultati:**

CRC pacijenti sa anemijom imali su težu i lokalno uznapredovalu bolest u poređenju sa CRC neanemičnim pacijentima. Anemija je pozitivno korelirala sa višom nuklearnom invazijom, invazijom limfe i krvnih sudova, kao i sa višim TNM stadijumom, uočljivim metastatskim lezijama u plućima i jetri i peritonealnoj karcinomatozi. Značajno veće koncentracije IL-33, Gal-1 i IL-1 pronađene su u serumima pacijenata sa CRC i otkrivenom anemijom. CRC pacijenti su uglavnom imali mikrocitnu anemiju, dok su vrednosti feritina bile u normalnom opsegu. Analiza je otkrila pozitivnu međusobnu korelaciju između serumskih vrednosti galektina-1, IL-1 i IL-33 kod pacijenata sa CRC. Nivo hemoglobina u negativnoj korelaciji sa serumskim IL-33, Gal-1 i IL-1. Analizirali smo ROC krive seruma IL-33, Gal-1 i IL-1 što je ukazalo da se ovi citokini mogu tretirati kao dodatni markeri za anemiju upale kod pacijenata sa CRC.

**Zaključak:**

Predominacija galektin-1, IL-1 i IL-33 kod anemičnih CRC pacijenata implicira na njihovu potencijalnu ulogu u nastanku anemije i daljem razvoju.

## Introduction

CRC is the second most often cause of death from malignancy in the world. Regarding women population, CRC is the second most frequent cancer and the third regarding men. There is a significant increase of the colorectal cancer incidence due to unhealthy lifestyles and ageing of world population [1]. The revised World Cancer Research Fund and American Institute for Cancer Research report that the high consumption of red meat and meat products, obesity and alcohol raise the possibility of CRC development, while on the other hand activity and exercising are protective factors [2].

Anemia is common in patients with CRC and can be one of the most often extraintestinal signs of CRC, which is present in 30%-75% of patients [3] [4]. Several studies have reported an association between anemia, both microcytic or normocytic, and poorer cancer specific survival of patients due to systemic inflammation and nutritional status [5] [6]. Anemia in CRC patients is often the consequence of chronic bleeding from the tumor; which can be occult from a right colon tumor or visible presence of blood in feces from left colon or rectal cancers [4]. However, anemia can also be caused during systemic inflammatory response to the tumor with higher concentrations of inflammatory cytokines such as interferon gamma (IFN-γ), different sorts of interleukins (IL-1, IL-6, IL-8 and IL-10) and tumor necrosis factor alpha (TNF-α) that directly or indirectly stimulate progression of anemia [3] [7].

IL-33, by biding to its receptor ST2, stimulates *in vivo* growth of CRC cells both in human and murine and their sphere formation and inhibits tumor apoptosis induced by chemotherapy [8]. Galectin-1 (Gal-1) is Galactoside-binding lectin with many functions, which is involved in different phases of tumorigenesis: stimulation of cell growth and migration, cells interactions, angiogenesis, tumor-immune escape [9]. IL-1 is produced by various cells, such as myeloid cells types, mostly after inflammatory or stress conditions [10]. Previous studies confirmed importance of IL-1 in different processes, for example genesis of tumor cells, their development and invasiveness, stimulation or inhibition of immune response against tumor [10]. The exact contribution of these mediators in pathological genesis of anemia in CRC patients has not been elucidated yet.

The main goal of this research has been to analyze Gal-1, IL-1 and IL-33 values both in anemic and non-anemic CRC patients.

## Methods

### Ethical statement

This study was performed in Clinical Center of Kragujevac (Gastroenterology Center) and Faculty of Medical Sciences (Center for Molecular Medicine and Stem Cell Research), University of Kragujevac, Serbia. All examined subjects gave informed consent. EthicsCommittees of the Clinical Center of Kragujevac,Serbia (Approval Number 01/1627) and Faculty ofMedical Sciences, University of Kragujevac, Serbia(Approval Number 01-311/6) have given ethicalapprovals. All research procedures were carried out inaccordance with the Declaration of Helsinki and thePrinciple of Good Clinical Practice.

### Patients

For the purpose of this research, 55 CRC patients were involved and examined. CRC was confirmed after endoscopic and histopathological examination. Patients with previous treatments (chemotherapy, radiation, antibiotics, immunosuppressive agents, salicylates, corticosteroids and biological therapy) or without adequate clinical documentation available were not included. Excluding criteria also were comorbidity with other gastroenterological diseases (esophageal reflux disease, ulcer disease, helicobacter pylori infection, malabsorptive syndrome, diverticulous bowel disease), autoimmune, inflammatory, malignant and infectious diseases or active bleeding. We collected and investigated clinical data about age, sex (determined with physical examination), cancer size, localization, presence of metastasis, invasion of vascular and lymphatic vessels, TNM clinical stage, histological differentiation rate and nuclear grade (using American Joint Committee on Cancer - AJCC classification from 7th edition in 2010).

### Measurement of serum Galectin-1, IL-33 and IL-1

Serum levels of cytokines of interest were measured, as previously described [11]. Shortly, 10 mL ofblood were taken from each examined subject at 8ambefore any therapy. Collected samples have beenstored at -80°C, until ELISA testing, which was performed according to the instructions of manufacturer (Systems R&D, Minneapolis, USA).

### Tumor markers measurement

Levels of tumor markers, such as carcinoembryonic antigen-CEA, alpha-fetoprotein-AFP and cancer antigen CA 19-9, in sera of each patient were evaluated in the laboratory of Clinical Center Kragujevac by CLIA methods (chemiluminescence enzyme immunoassay).

### Statistical analysis

With SPSS software (20.0) we have performed all data analyses. Our results were presented as (mean ± standard error). To determine statistical significance between the means of two groups we used Mann-Whitney U-test or Student's t-test, where adequate and for the correlation between the markers of interest with anemia in CRC patients, we used Pearson's or Spearman's correlation, also where adequate. We used the Chi Square Test to evaluate the statistical significance of data presented in tables. Statistically significant p-values were for p ≤ 0.05.

## Results

All 55 adult patients diagnosed with CRC have been included in this research (27 without anemia, 28 with anemia). Clinico-pathological features of all patients are shown in Table 1. The analysis of the data also did not show any significant difference regarding the distribution of age and gender.

**Table 1:** Clinico-pathological characteristics of examinedpatients

Characteristics	Colorectal carcinoma (CRC) patients	
	Without anemia	With anemia
Sex (male/female)	17/10	16/12
Age (mean (range))	63.4 (50–75)	66.5 (53–82)
Hemoglobin, g/L	138.04 ± 9.33	96.77 ± 4.34
MCV	89.36 ± 11.09	70.77 ± 6.63
Platelets (x10^9^/L)	314.23 ± 12.46	408.63 ± 29.55
Histological differentiation rate (well/moderate)	6/21	7/21
AFP	3.26 ± 1.64	238.84 ± 128.30
CEA	33.79 ± 22.25	223.14 ± 109.11
CA 19-9	81.57 ± 70.51	931.07 ± 190.05

### CRC patients with anemia have severe and local advanced disease

**Figure 1:**
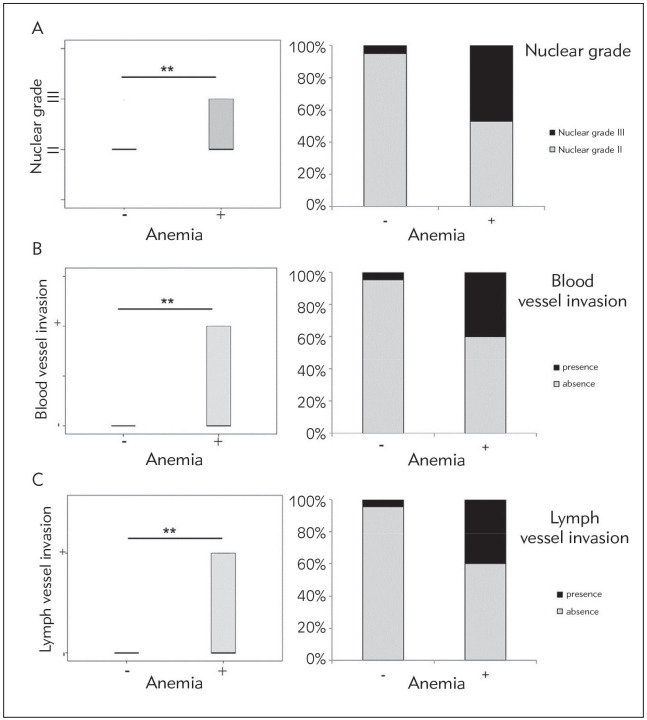
CRC patients with anemia have severe and local advanced disease A. Patients with CRC and anemia had higher nucleargrade of colorectal cancer<br>B. Higher percentage of blood vessels invasion or<br>C. lymph vessels invasion in comparison to CRC patients without anemia<br>Chi-Square test was used (* p < 0.05; ** p < 0.01)

We have formed two groups according to the hemoglobin concentration (with and without anemia). This classification was based on the presence of anemia, which is diagnosed when hemoglobin concentration is less than 130 g/L in male patients and less than 120 g/L in female patients. Evaluation of nuclear grade of colorectal cancer was made in patients with the presence or absence of anemia, using AJCC classification [12]. According to nuclear grade all CRC patients have been split into groups I, II and III. Results have shown that anemic patients had significantly higher nuclear grade of colorectal cancer in comparison to non-anemic patients (p = 0.003; Figure 1A). Moreover, according to the evidence of detectable invasion of blood or lymph vessels we have split all CRC patients into groups (+ or-). Significantly higher percentage of CRC patients with diagnosed anemia had detectable invasion of blood and lymph vessels in comparison to CRC patients without anemia, respectively (p = 0.009; p = 0.008; Figure 1B, Figure 1C ). Further, anemia positively correlated with higher nuclear grade (r = 0.489; p = 0.002), lymph (r = 0.444; p = 0.006) and blood vessel invasion (r= 0.439; p = 0.007).

### Anemia correlates with more progressive form of CRC

**Figure 2:**
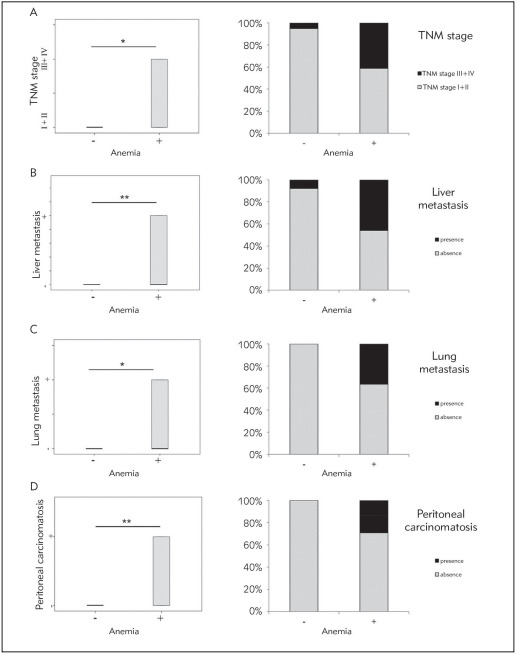
Anemia correlates with more progressive form of CRC A. Patients with CRC and anemia had significantly advancedTNM stage<br>B. higher percentage of detectable metastatic lesions in liver<br>C. lung or<br>D. peritoneal carcinomatosis compared to CRC patients without anemia<br>Chi-Square test was used (* p < 0.05; ** p < 0.01)

According to TNM clinical stage, we have split all CRC patients into two groups (I+II or III+IV). CRC patients with anemia had significantly advanced TNM stage (or had TNM score III or IV), while CRC patients without anemia (or with normal level of hemoglobin) mostly had TNM stage I or II (p = 0.011; Figure 2A). According to the evidence of liver metastasis, lung metastasis or peritoneal carcinomatosis, we have split all CRC patients into groups (+ or-). Higher percentage of CRC patients with anemia had detectable metastatic lesions in liver and lung as well as peritoneal carcinomatosis, respectively, compared to CRC patients without anemia (p = 0.003; p = 0.031; p = 0.004; Figure 2B, Figure 2C, Figure 2D). Additionally, anemia positively correlated with higher TNM stage (r = 0.431; p = 0.009), detectable metastatic lesions in lung (r = 0.422; p = 0.019) and liver (r = 0.428; p = 0.002) and peritoneal carcinomatosis (r = 0.417; p = 0.003), respectively. Further, tumor markers, such as CEA, AFP and CA 19-9 have been significantly higher in patients with CRC and anemia incomparison to patients with CRC without anemia (Table 1). Moreover, results have shown that hemoglobin negatively correlates with tumor markers CEA(r = -0.475; p = 0.001), AFP (r = -0.383; p = 0.007) and CA 19-9 (r = -0.610; p = 0.001), respectively. Negative correlations were also detected between MCV and CEA (r = -0.343; p = 0.020), AFP (r = -0.277;p = 0.049) and CA 19-9 (r = -0.369; p = 0.014), respectively.

### Patients with CRC and anemia had increasedIL-33, Gal-1 and IL-1 values in serum

**Figure 3:**
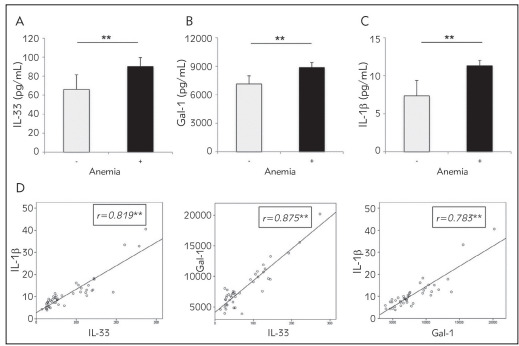
A, B, C Anemic CRC patients had higher concentration of IL-33, Gal-1 and IL-1 b in serum compared to non-anemic CRC patients<br>D. Positive correlation between galectin-1 and IL-33, galectin-1 and IL-1 and IL-33 and IL-1 values in serum of CRC patients<br>Correlation was determined using Spearman’s test (* p< 0.05; ** p< 0.01)

Serum values of cytokines of interest were analyzed in serum of CRC patients with/withoutdiagnosed anemia. Significantly higher concentrationof IL-33 (p = 0.003), Gal-1 (p = 0.009) and IL-1 (p= 0.005) have been found in serum of CRC patientswith detected anemia (Figure 3A, Figure 3B, Figure 3C). Positive correlation was detected between CA 19-9 and IL-1(r = 0.321; p = 0.029) and IL-33 (r = 0.438; p =0.002), respectively. Moreover, analysis revealed positive relation between serum values of galectin-1 and IL-33 (r = 0.772; p = 0.001), galectin-1 and IL-1 (r= 0.828; p = 0.001) and IL-33 and IL-1 (r = 0.879;p = 0.001) in CRC patients (Figure 3D).

### CRC patients mostly had microcytic anemia

**Figure 4:**
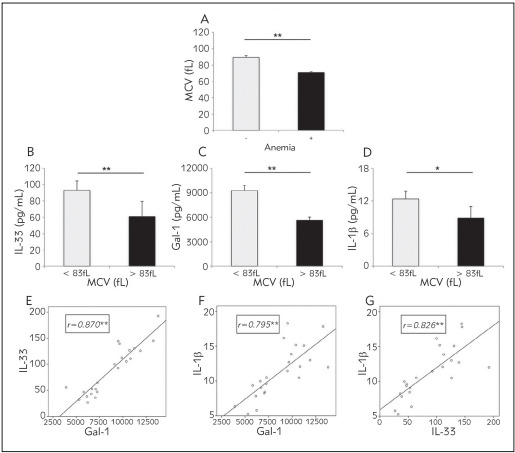
A Significantly lower MCV of red blood cells in anemic CRC patient<br>B, C, D. Significantly higher IL-1, Gal-1 and IL-1β in serum of CRC patients with microcytic anemia in comparison to CRC patients with normocytic anemia or without anemia<br>E,F, G.Strong positive correlation was detected between galectin-1 and IL-33, galectin-1 and IL-1 β and IL-33 and IL-1 β, valuesin serum of CRC patients with diagnosed microcytic anemia<br>Correlation was determined using Spearman’s test (* p < 0.05; **p < 0.01)

Further, analysis of the volume of red blood cells (mean corpuscular volume - MCV) was made in CRC patients with and without anemia. MCV has been significantly lower in patients with CRC and detected anemia in comparison to patients CRC with normal hemoglobin level (p = 0.001; Figure 4A). In order to confirm previous results, we have formed two groups of CRC patients according to the MCV values and analyzed IL-33, Gal-1 and IL-1 values in serum. CRC patients with microcytic anemia (MCV < 83fL) had significantly higher IL-33 (p = 0.009), Gal-1 (p = 0.002) and IL-1 (p = 0.013) in sera in comparison to CRC patients with normocytic anemia or without anemia (Figure 4B, Figure 4C, Figure 4D). Additionally, strong positive relation has been detected in comparison of galectin-1 and IL-33 (r = 0.870; p = 0.001), galectin-1 and IL-1 (r = 0.795; p = 0.001) and IL-33 and IL-1 (r = 0.826; p = 0.001) values in serum of CRC patients with diagnosed microcytic anemia (Figure 4E, Figure 4F, Figure 4G). Further, we measured ferritin in all patients. Ferritin values were in normal range and without any significant difference in ferritin levels when comparing anemic CRC patients to non-anemic CRC patients (data not shown).

### Thrombocytosis correlates with anemia

**Figure 5:**
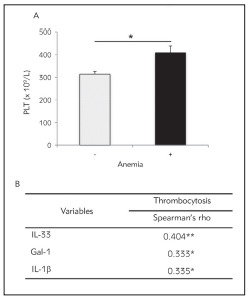
A Patients with CRC and anemia had significantlyhigher number of platelets compared to non-anemic patientswith CRC<br>B. Positive correlation between thrombocytosisand IL-33, Gal-1 and IL-1 b, respectively. Correlation wasdetermined using Spearman’s test (* p < 0.05; ** p < 0.01)

Additional blood analysis revealed significantly higher number of platelets in CRC patients with detected anemia compared with non-anemic CRC patients (p = 0.03; Figure 5). Thrombocytosis has positive correlation with IL-33 (r = 0.404; p = 0.004), Gal-1 (r = 0.333; p = 0.024), IL-1 (r = 0.335; p = 0.020) values in serum, respectively (Figure 5).

### IL-33, Gal-1 and IL-1 concentrations negatively correlate with hemoglobin

**Figure 6:**
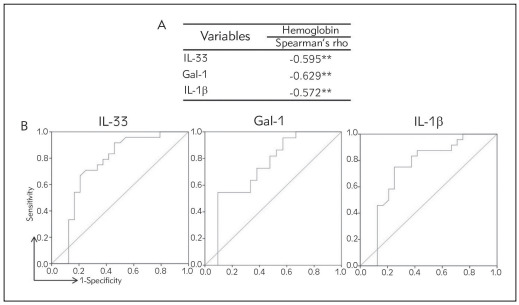
A Moderate negative correlation between serum hemoglobin level and IL-33, Gal-1 and IL-1 b, respectively<br>B. Specificity and sensitivity of IL-33, Gal-1 and IL-1 in serum<br>Correlation was determined using Spearman’s test (* p < 0.05;** p < 0.01)

Finally, we have tested correlation of serum level of hemoglobin with IL-33, Gal-1 and IL-1 values in serum. The results have shown moderate negative cor relation between hemoglobin and serum IL-33 (r =-0.595; p = 0.001), Gal-1 (r =-0.629; p = 0.001) and IL-1 (r =-0.572; p = 0.001), respectively (Figure 6). Examination of ROC curves of IL-33, Gal-1 and IL-1 values in serum showed that these cytokines could predict anemia in CRC patients (Figure 6). Our data demonstrate that IL-33 (sensitivity 95.8%, specificity 45.8%) Gal-1 (sensitivity 59.1%, specificity 66.7%) and IL-1 (sensitivity 75%, specificity 75%) can be potential markers for paraneoplastic anemia in CRC patients. The optimal cut off levels of cytokines estimated for confirmation of anemia in CRC patients was 31.58 pg in mL (IL-33), 7257.07 pg in mL (Gal-1), 8.9624 pg in mL (IL-1).

## Discussion

In this present research, we have shown that CRC patients with anemia had significantly higher nuclear grade in comparison to CRC non-anemic patients (Figure 1). CRC patients with diagnosed anemia had significantly higher TNM stage as well as invasion of lymph and blood vessels and detectable metastatic lesions in lung and liver and peritoneal carcinomatosis compared to CRC non-anemic patients (Figure 2). Higher concentrations of CEA, AFP, CA 19-9 were detected in patients with CRC and detectable anemia (Table 1). Tumor markers negatively correlated with hemoglobin and MCV. As previous study confirmed positive correlation between tumor markers and CRC progression, our data implicate on more severe form of disease in anemic patients [13]. Our finding of severe and more progressive disease in anemic CRC patients are in line with many previous studies [14]. As anemia is common in patients with CRC and can be one of the most often extraintestinal signs of CRC, we have measured MCV in these patients to confirm the type of anemia [3]. Analyses have confirmed significantly lower levels of MCV in anemic patients with CRC in comparison to non-anemic patients with CRC. According to these results, it is possible that predomination of microcytic anemia in CRC patients suggests on possible gastrointestinal bleeding. However, normal range of ferritin values in CRC patients with anemia implicate on other causes of detected anemia, but bleeding. According to Moreno et al. [15] decreased MCV with normal ferritin values implicate on chronic disease anemia. Anemia can also be a consequence of systemic inflammatory response in presence of tumor [3]. Cancer related anemia occurs without bleeding and results from chronic inflammation and synthesis of pro-inflammatory cytokines simultaneously by different kinds of cells, both immune and cancer cells [16]. The synthesized pro-inflammatory cytokines can cause anemia by different pathological mechanisms [7]. They stimulate hepatic production of hepcidin. Hepcidin is known as an inhibitor of iron absorption in the duodenum. Further, cytokines can facilitate iron storage in macrophages. This retention of iron in macrophages causes limited availability of iron for erythroid cells. Moreover, previous studies showed that cytokines with proinflammatory activities can have a direct inhibitory influence on erythroid progenitors' proliferation and differentiation or they can activate macrophages and thus increasing the process of erythrophagocytosis [3] [7].

As previous studies clearly confirmed the importance of proinflammatory cytokines in biology of colorectal cancer [3] [7], our research was further based on the investigation of the systemic concentrations of proinflammatory cytokines. IL-1 is a pleiotropic cytokine involved in the few processes in tumorigenesis such as tumor growth, metastasis, and angiogenesis [10]. IL-1 can also act indirectly via stimulation of production of matrix metalloproteinases and different kinds of cytokines, for example IL-6, IL-8, TNFα, TGF and VEGF [17]. Our study showed significantly higher IL-1 concentration in patients with CRC and detected anemia compared to non-anemic CRC patients as well as positive association between CA 19-9 and IL-1 and negative association between hemoglobin and IL-1 (Figure 3, Figure 6). Previous studies are in line with our results suggesting a significant role of IL-1 in the development and pathological genesis of anemia. IL-1 affects iron metabolism, thus disabling utilization of iron for synthesis of hemoglobin [17]. IL-1 decreases level of erythropoietin in vitro, modulates erythropoietin receptors and also inhibits production of erythroid progenitors [18].

Interleukin-33 (IL-33) is a member of IL-1 cytokine family. Its role in immune response regulation after cellular stress or damage is crucial [19] and also is involved in pathogenesis of CRC development. IL-33 has many functions, for example in host immunity against tumor as an inhibitor, in angiogenesis as a promoter and can also induce tumor stroma remodeling. It can also affect the activation of NF-kB trans criptional factor; stimulate production of proinflammatory cytokines thereby promoting inflammation [20]. Our result revealed significantly higher systemic IL-33 concentration in anemic CRC patients, positive association between CA 19-9 and IL-33 and negative association between hemoglobin and IL-33 (Figure 3, Figure 6), suggesting on possible role of this proinflammatory cytokine in pathogenesis of anemia in CRC patients. Other study showed that IL-33 is highly expressed in endothelial cells thus promoting the expansion of different kind of hematopoietic precursors and regulating myelopoiesis *in vitro* and *in vivo*, implicating its role in hematopoiesis [21] [22]. Stankovic et al. [23] revealed that during acute inflammation IL-33/ST2 axis plays an essential role in metabolism of Fe, mainly by increasing concentration of iron at the place of inflammation and thus decreasing blood mean corpuscular hemoglobin. The most often cause for development of microcytic anemia is iron deficiency, while on the other hand iron sequestration facilitated by IL-1 and IL-33 might be an alternative cause of anemia in CRC patients.

Galectin-1 in CRC exhibits different aspects of tumor progression, such as cell adhesion, tumor cell transformation and growth. Also, it enhances migration of cancer cells, their proliferation and metastasis, inhibition of apoptosis and stimulation of angiogenesis [24] [25]. During the progression of CRC, galectin-1 is often significantly changed, meaning that overexpression of galectin-1 [24] correlates with poorly differentiated, invasive form of CRC with lymph node metastasis and shorter patient survival [25]. Our previous study has also shown importance of Galectin-1 in the pathology of CRC. We noticed fecal Gal-1 in feces samples of patients with more advanced form of CRC [11]. Moreover, Gal-1 positively correlates tumor markers, such as AFP and CA 19-9 and with tumor histological differentiation stage [11]. According to available information, this is the first research, which investigates connection between systemic Gal-1 and anemia in CRC patients. In our study, we showed that patients with CRC and detected anemia had significantly higher level of systemic Galectin-1 compared to the group of CRC patients without anemia (Figure 3). Moreover, Galectin-1 negatively correlated with hemoglobin (Figure 6). IL-1 and IL-33 are members of the same IL-1 family of cytokines and thus share many effector functions [10] [19]. Galectin-1 and IL-33 share the same tumor promoting effect in CRC, by helping tumor cells in bypassing of apoptosis [8] [26] [27]. It is possible that Gal-1 through NK-B signaling pathway promotes proinflammatory cytokines production, such as IL-1 production and subsequently attenuates hematopoiesis.

**Figure 7:**
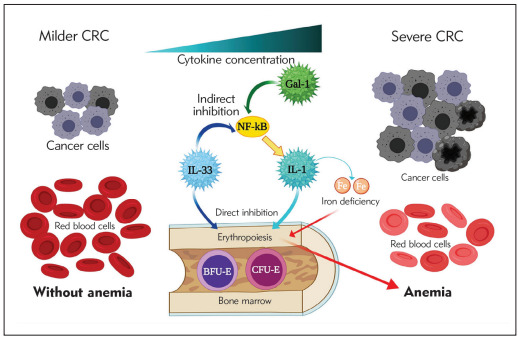
Potential synergistic effect of IL-1 and IL-33 in pathogenesis of anemia of inflammation in CRC patients 1. IL-1 and IL-33 directly inhibit erythropoiesis and disable synthesis of hemoglobin via suppression of iron utilization<br>2. Gal-1 and IL-33 activate NF-kB transcriptional factor and indirectly stimulate proinflammatory cytokines production, such as IL-1 production, which then inhibits erythropoiesis. In turn subsequent anemia correlates with severe and more progressive CRC<br>(*Created by the authors with BioRender.com)

Revelation of positive correlation between systemic IL-1, IL-33 and galectin-1 levels in serum of anemic CRC patients suggested on synergistic effect of these cytokines in pathogenesis of anemia in CRC patients. Based on previous data, it is believed that IL-1 and IL-33 directly disable synthesis of hemoglobin via suppression of iron utilization, while indirectly Gal-1 and IL-33 activate NF-kB transcriptional factor and stimulate proinflammatory cytokines production, such as IL-1 production that in turn inhibits erythropoiesis, as described in Figure 7. Further, examination of ROC curves of Gal-1, IL-33 and IL-1 revealed that these cytokines could predict anemia of inflammation in CRC patients (Figure 6).

Reactive thrombocytosis, an elevated platelet, can be caused due to deficiency of iron, infection (acute or chronic) and different inflammatory diseases. Nowadays, cancers are frequently connected with paraneoplastic thrombocytosis. Thrombocytosis often accompanies cancer growth and metastatic dissemination [28]. Recent studies revealed possible association between thrombocytosis and poor prognosis for patients with CRC [29]. Paraneoplastic elevation of platelet count is associated with worse overall survival, as well as cancer-specific and disease-free survival in CRC patients. Moreover, there are strong evidence regarding significant correlation between tumor location, higher TNM clinical stage (T3-4), presence of metastasis or lymph node and vessels invasion, and also poor histological differentiation with thrombocytosis in CRC patients [30] [31]. In our study, we noticed significantly higher number of platelets in the group of CRC patients with anemia in comparison to the group of non-anemic CRC patients (Figure 5). Systemic values of cytokines Gal-1, IL-1 and IL-33 positively correlated with higher platelets number (Figure 5). Previous study claimed that IL-1 stimulates megakaryopoesis thus increasing the number of platelets [32]. The other study showed that mice with over expression of IL-33 had detectable anemia and thrombocytosis [22]. Possible explanation for thrombocytosis and positive correlation between higher number of platelets and mediators of interest is that enhanced levels of IL-1 and IL-33 could directly induce megakaryopoiesis and subsequently thrombocytosis.

## Conclusion

Presented data revealed higher Galectin-1, IL-1 and IL-33 values in serum of CRC patients with anemia, establishing them as new players in genesis and progression of anemia of inflammation (Figure 7). These findings point on mechanism of anemia genesis in CRC patients, besides occult bleeding, that deserves further clarification.

## Dodatak

### Acknowledgements:

The Serbian Ministry of Education, Science and Technological Development (175069 and 175071), project with PR China(06/2018) and the Faculty of Medical SciencesKragujevac (projects JP04/15, JP15/19 andJP11/18), Serbia, have supported this study.

### Conflict of interest statement

All the authors declare that they have no conflictof interest in this work.
